# Combining indoor and outdoor methods for controlling malaria vectors: an ecological model of endectocide-treated livestock and insecticidal bed nets

**DOI:** 10.1186/s12936-017-1748-5

**Published:** 2017-03-13

**Authors:** Laith Yakob, Mary Cameron, Jo Lines

**Affiliations:** Department of Disease Control, The London School of Hygiene & Tropical Medicine, Keppel Street, London, WC1E 7HT UK

## Abstract

**Background:**

Malaria is spread by mosquitoes that are increasingly recognised to have diverse biting behaviours. How a mosquito in a specific environment responds to differing availability of blood-host species is largely unknown and yet critical to vector control efficacy. A parsimonious mathematical model is proposed that accounts for a diverse range of host-biting behaviours and assesses their impact on combining long-lasting insecticidal nets (LLINs) with a novel approach to malaria control: livestock treated with insecticidal compounds (‘endectocides’) that kill biting mosquitoes.

**Results:**

Simulations of a malaria control programme showed marked differences across biting ecologies in the efficacy of both LLINs as a stand-alone tool and the combination of LLINs with endectocide-treated cattle. During the intervals between LLIN mass campaigns, concordant use of endectocides is projected to reduce the bounce-back in malaria prevalence that can occur as LLIN efficacy decays over time, especially if replacement campaigns are delayed. Integrating these approaches can also dramatically improve the attainability of local elimination; endectocidal treatment schedules required to achieve this aim are provided for malaria vectors with different biting ecologies.

**Conclusions:**

Targeting blood-feeding mosquitoes by treating livestock with endectocides offers a potentially useful complement to existing malaria control programmes centred on LLIN distribution. This approach is likely to be effective against vectors with a wide range of host-preferences and biting behaviours, with the exception of species that are so strictly anthropophilic that most blood meals are taken on humans even when humans are much less available than non-human hosts. Identifying this functional relationship in wild mosquito populations and ascertaining the extent to which it differs, within as well as between species, is a critical next step before targets can be set for employing this novel approach and combination.

**Electronic supplementary material:**

The online version of this article (doi:10.1186/s12936-017-1748-5) contains supplementary material, which is available to authorized users.

## Background

The burden of malaria has been reduced considerably in the last 15 years following sustained, large-scale mosquito control efforts [1]. However, the continued success of vector control programmes is threatened by several concomitant factors. The biggest threat has been the emergence and rapid spread of insecticide resistance among the principal vector species. Resistance exists to all classes of chemical insecticide currently endorsed by the World Health Organization (WHO) for mosquito control, with numerous mosquito populations across sub-Saharan African and Asia demonstrating resistance to multiple chemical classes at once [2, 3]. Behavioural changes have also been observed among malaria vectors whereby species (or sibling species) that were previously inclined to bite people indoors during sleeping hours now bite at different times of the day and/or bite outdoors [4–6]. Despite being a long-recognized threat to malaria vector control [7, 8], it remains unclear whether this change is phenotypic or genetic. Additionally, the proportional composition of overlapping mosquito species (or sibling species) has drastically shifted in recent years to favour mosquitoes that have always been more inclined to bite outdoors and that are less discerningly anthropophagic [9]. An accelerated research effort to develop alternative control methods that are not attenuated in their effect by extant resistance or behavioural resilience is strongly advocated.

One such development is the treatment of livestock with insecticides. Although harmless to the animals themselves, these ‘endectocides’ kill mosquitoes following the blood meal. The treatment of livestock with pour-on insecticides was first widely used for the control of ticks [10] and then tsetse flies [11]. Recently, there has been increased interest in the use of ivermectin: this is an anthelminthic used at large scale for the control of river blindness in humans and veterinary diseases in livestock. Its function of killing mosquitoes on ingestion is coincidental and was first observed only recently [12]. A shortcoming of ivermectin for mosquito control is the rapidity with which it is metabolized, which means frequent application would be needed [13]. However, a study by Poche et al. [14] recently demonstrated highly effective mosquitocidal properties of diflubenzeron, eprinomectin and fipronil when topically or orally administered to cattle. They demonstrated significant mortality (50%) among mosquitoes within 2 days of feeding on cows after up to 1 month of being orally treated with 1 mg/kg fipronil.

The next stage for assessing the suitability of this approach for controlling malaria vectors is to identify when and where its implementation would have maximal benefit as part of an integrated vector management strategy together with the current malaria control mainstay of long-lasting insecticidal nets (LLINs). Increasingly, mathematical models have become integral components of formulating infectious disease control strategy [15], especially so in the context of malaria because of the long history this analytic method has had in describing *Plasmodium falciparum* transmission [16]. Here, a mathematical model adapted from [17] is described and used to project the anticipated malaria control efficacy of complementing LLINs with endectocides. Simulations are designed to account for a diverse range of mosquito-biting behaviours.

## Methods

### Biting ecology

For endectocide-treated livestock to be considered a feasible approach to malaria control, key malaria vector species must be demonstrated to source a non-negligible proportion of their blood meals from these animals. *Anopheles gambiae* sensu stricto are widely regarded as the paragons of anthropophagy among vectors of human disease, but even these notorious human-biters have repeatedly been found to source between 10 and 30% of their blood from cattle [18–21]. Moreover, these blood meal-analysis studies typically focus on vectors caught indoors and thereby bias their estimates towards human-biting [7]. The other major African malaria vector, *Anopheles arabiensis*, is even less discerning in its host choice and proportionally more meals have been identified to originate from cattle from field-caught mosquitoes [22, 23]. A comprehensive review recently emphasized that most malaria vector species globally are zoophagic [24]. For all of these species, wide ranges in the human blood index (HBI) have been described. Therefore, it is likely that endectocidal efficacy will be driven not only by intrinsic host-preference of mosquito (sibling) species but also by extrinsic factors affecting availability of different sources of blood. A simple function that describes a range of biting behaviours resulting from this mix of intrinsic and extrinsic factors is:

1 $$p_{H} = \frac{{\dot{H}}}{{\dot{H} + \alpha (1 - \dot{H})^{\beta } }} .$$

In the above function, the proportion of bites on humans (*p*_*H*_) is determined by the availability of humans relative to all potential blood sources, $\dot{H}$; but also shaped by parameters *α* and *β* (whereby *α* and *β* > 0). Here, it is assumed that sources of blood other than humans and cattle are negligible. This function was inspired by, and adapted from, what is referred to in the ecological literature as the Holling’s Type functional responses [25, 26]. The different Types are associated with qualitatively distinct relationships between the availability of a resource and the rate at which that resource is consumed. At its simplest, there is direct linear proportionality: doubling the availability of a resource doubles the rate at which it is consumed. This is known as a Type I response. This is modelled in the above function by setting *α* and *β* to equal 1. Many previous models have allowed for fixed differences between alternative host species [27–30], and this is achieved by keeping *β* equal to 1 and changing the value of *α*. For example, when α equals 0.5, a vector that has half the preference for cattle compared with humans is modelled. Critically, in these previous formulations, this fixed intrinsic preference was limited to depicting a linear relationship between the HBI and the availability of human hosts relative to all hosts. Decades of empirical studies exploring how species consume their resources has yielded only one example of a Type I response (for mollusc bivalves), the rest (including all arthropod studies) exhibit more complex, non-linear relationships [31]. Data for blood-feeding disease vectors do not yet exist. The flexibility of the above function was exploited to explore the impacts of several non-linear responses of vectors to the availability of hosts. When *α* < 1 and *β* > 1, a convex-up relationship forms between HBI and human availability (relative to all blood hosts) and this has been referred to as a Type II response; *α* > 1 and *β* > 1 results in a saturating sigmoidal curve (Type III); *α* > 1 and *β* < 1 results in a convex-down form (Type IV); and, *α* < 1 and *β* < 1 generates a reverse sigmoidal curve (Type V). These different functional Types are illustrated in the first column sub-plots of the figures. Their biological interpretations are described in Table 1 and detailed further in [32].

**Table 1:** The qualitatively different behavioural responses (parameterization and associated vector behaviours) described by the new formula (adapted from [46])

Response Type	Ecological equivalent	Parametric conditions	Vector behaviour
Type I	Holling’s Type I	α = 1 β = 1	Indiscriminate, or vector biting that is consistent (proportionate) across relative availabilities of alternative hosts
Type II	Holling’s Type II	α < 1 β ≥ 1	An anthropophilic vector which takes most of its blood meals on humans even when humans are less available than other hosts, and when humans and non-humans are equally available, almost all blood meals are taken from humans
Type III	Holling’s Type III	α ≥ 1 β > 1	This is the pattern expected with a learned behaviour, such that female mosquitoes learn to prefer the more common Type of host
Type IV	Inversion of Holling’s Type II	α > 1 β ≤ 1	A zoophilic vector is disinclined to bite humans until they constitute all but the only available blood source
Type V	Inversion of Holling’s Type III	α ≤ 1 β < 1	HBI saturates and becomes relatively invariant when humans and non-humans are at similar availability. This is analogous to ‘negative prey switching’ whereby the ‘predator’ consumes disproportionately less of the more available ‘prey’ [45]. Eventually, when non-humans become vanishingly rare, the HBI is forced to increase sharply to unity

### Epidemiological model

**Fig. 1:**
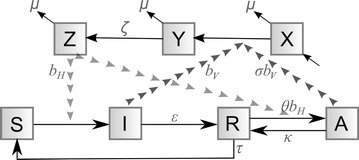
The compartmental framework of the epidemiological model. Humans are either susceptible (*S*), infected (*I*), recovered (*R*) or asymptomatic infected (*A*). Mosquitoes are either susceptible (*X*), infected but not yet infectious (*Y*) or infectious (*Z*). Infectious humans infect susceptible mosquitoes (*dark arrow tracks*) and infectious mosquitoes infect susceptible or recently recovered humans (*light arrow tracks*). Full details of parameter definitions can be found in Table 2

The biting function was then incorporated into a simple epidemiological model of malaria infection dynamics:

2 $$\frac{dI}{dt} = mp_{H} b_{H} SZ - \left( {\varepsilon + \mu_{H} } \right)I$$

3 $$\frac{dR}{dt} = \varepsilon I + \kappa A - \left( {\theta mp_{H} b_{H} Z + \tau + \mu_{H} } \right)R$$

4 $$\frac{dA}{dt} = \theta mp_{H} b_{H} RZ - \left( {\kappa + \mu_{H} } \right)A$$

5 $$\frac{dY}{dt} = p_{H} b_{V} \left( {I + \sigma A} \right)X - \left( {\zeta + \mu_{V} } \right)Y$$

6 $$\frac{dZ}{dt} = \zeta Y - \mu_{V} Z$$

where by humans are Susceptible (*S*), Infected (symptomatic, ‘*I*’), Recovered (*R*) or Asymptomatically infected (*A*); and a stable population was assumed: *S* = 1−  (*I* + *R* + *A*). Infections were split between symptomatic and asymptomatic infections because a disease burden-reduction campaign may have different priorities than an elimination campaign, e.g., the former may target reducing ‘*I*’ whereas the latter may want to reduce both ‘*I*’ and ‘*A*’. It is assumed that an asymptomatic infection can only arise in an individual that has recently been infected already. (Figure 1 shows the compartmental framework for the epidemiological model). This is a simplistic representation of immunity in accordance with many other contemporary models of malaria [33]. If the projected impacts of endectocide-treated cattle on malaria immunity in humans were to be investigated, a significantly more complex model structure would be required.

Following convention of standard mathematical models of malaria, m denotes the ratio of mosquitoes to humans; *b* denotes the transmission coefficient (subscript ‘*H*’—from vector to host; subscript ‘*V*’ from host to vector) and is calculated as the maximum daily bite rate (1/3 i.e., one bite per three days) multiplied by the probability of parasite transmission (see Table 2); *μ* denotes the mortality rate (subscript ‘*H*’ for humans; subscript ‘*V*’ for vectors). Additionally, *ε* and *κ* are the respective rates of recovery from symptomatic and asymptomatic infection; *τ* is the rate of loss of immunity; and *θ* allows for a differential susceptibility of recovered individuals to secondary infection. Mosquitoes are susceptible (*X*), incubating (*Y*) or infectious (*Z*); and a stable mosquito population is assumed: *X* = 1 − (*Y* + *Z*). *σ* allows for a different level of parasite transmissibility from asymptomatic infected individuals to vectors (relative to symptomatic individuals); and *ζ* is the reciprocal of the extrinsic incubation period for the parasite. Parameter definitions and sources for their values are described in Table 2.

**Table 2:** Model parameter definitions, values and sources

	Definition	Value [cited literature]
*b*_*H*_	Transmission coefficient (vectors → hosts) = bite rate (day^−1^) × transmission probability	0.1 = 1/3 × 0.3 [47]
*b*_*V*_	Transmission coefficient (hosts → vectors) = bite rate (day^−1^) × transmission probability	0.007 = 1/3 × 0.02 [48]
*m*	Ratio of vectors to humans	Varied in simulations; values in figure legends
*ε*	Clearance rate of symptomatic infection (day^−1^)	1/200 [49]
*κ*	Clearance rate of asymptomatic infection (day^−1^)	1/200 [49]
*θ*	Level of reduced susceptibility to secondary infection	0.5 (assumed)
*τ*	Full susceptibility reversion rate (day^−1^)	1/1000 [50]
*μ*_*H*_	Birth and death rate of humans (day^−1^) (i.e., stable population)	1/21,900 (assumed)
*μ*_*V*_	Birth (or maturation) and death rate of vectors (day^−1^) (i.e., stable population)	1/10 [51]
*σ*	Adjustment factor for asymptomatic transmissibility to vector	0.25 [52]
*ζ*	Rate of parasite development within vector (day^−1^)	1/10 [53]

### Simulated control

Long-lasting insecticidal nets have the dual function of reducing the bite rate on humans and killing mosquitoes that come into contact with the insecticide with which they are treated. Both of these effects wane over time as the net accumulates holes and the insecticide loses potency. Recent studies with modern LLINs suggest a range of efficacy half-lives with a median value of approximately 2 years (i.e., after this period, the efficacy of protecting humans and in killing mosquitoes is 50% the original rate of a brand new net) [34, 35]. Similarly, estimates of initial efficacy of LLINs, in terms of mosquito mortality and personal protection, differ between studies. Universal (100%) coverage of a net that is (initially) 75% effective in personal protection and that results in 50% mosquito mortality within one day of contact fall within the reported range and are assumed here [36, 37]. To ensure sustained protection, current WHO guidelines recommend replacement of LLINs every three years. In practice, however, an interval of four years is probably more common, and so this is the frequency assumed in simulations.

Studies describing longitudinal endectocidal activity are scant; however, the study of Poche et al. [14] describe a maximum of 100% mosquito mortality following ingestion of blood from newly treated cattle and a half-life of between 21 and 28 days for 1.5 mg/kg oral fipronil. Here, a conservative estimate of 21 days is simulated. A wide range of different frequencies and coverage levels of endectocide are simulated in order to provide the first estimates of target levels with which to complement current policy goals for LLIN coverage.

Both the reduced rate in human-biting and the increased rate of mosquito mortality that results from simulated use of LLINs are assumed to exponentially decay in efficacy over time. Similarly, the increased mosquito mortality resulting from bites on livestock that are treated with endectocides is assumed to exponentially decay over time. Hence, *b*_H_* = *b*_*H*_ × [1 − *C*_*1*_ × (1 − ln(2)/730)^*t*^] denotes the diminished transmission potential from vector to human resulting from a reduced bite rate through LLINs (with an equivalent expression for *b*_*V*_); and *µ*_V_* = *µ*_*V*_ + 1/3 × [*p*_*H*_ × *C*_*1*_ × (1 − (ln(2)/730))^*t*^ + **(1** **−** ***p***_***H***_ **)** **×** ***C***_***2***_ **×** **(1** **−** **(ln(2)/21))** ^***t***^]. Control efficacy on mosquito mortality is a product of 1/3 because it is assumed that these vectors bite at a maximum of every three days on average. The square bracket in the equation describing mosquito mortality contains the increased mortality that comes about through contact with LLINs (the first half of the expression that is a product of *C*_*1*_: coverage—here ‘1’—multiplied by maximum efficacy of a new LLIN—here ‘0.75’) as well as through biting an endectocide-treated animal (the bold-font second half of the expression that is a product of *C*_*2*_: coverage—a full range from ‘0’ to ‘1’ is explored—multiplied by maximum efficacy of newly applied endectocide—here ‘1’). ‘*t*’ denotes the time-point (in days) after control distribution, i.e. allows for a decay in efficacy of the controls over time. With each round of newly distributed LLINs, any remaining control impact from prior rounds of LLINs are zeroed. Similarly for endectocidal applications. This simplification will only act to produce more conservative estimates in efficacy (for example, because some livestock that are not treated in the current round may have remaining endectocide).

For direct comparison of control efficacy across the different mosquito biting Types, baseline (pre-control) infection level was standardized at 50% malaria prevalence. This was achieved through adjustment of the vector-to-human ratio, *m*, until each control scenario was initiated under the same prevalence level. The alternative was to maintain a constant *m* but allow for different initial infection prevalence between the different scenarios of vector-biting behaviour; but it was deemed preferable to standardize models according to the more epidemiologically relevant metric. Details of precise *m* values needed to generate 50% prevalence are provided in the legends of the associated results plots. The model was implemented in Berkeley Madonna software using the Runge–Kutta 4 method of numerical analysis.

## Results

**Fig. 2:**
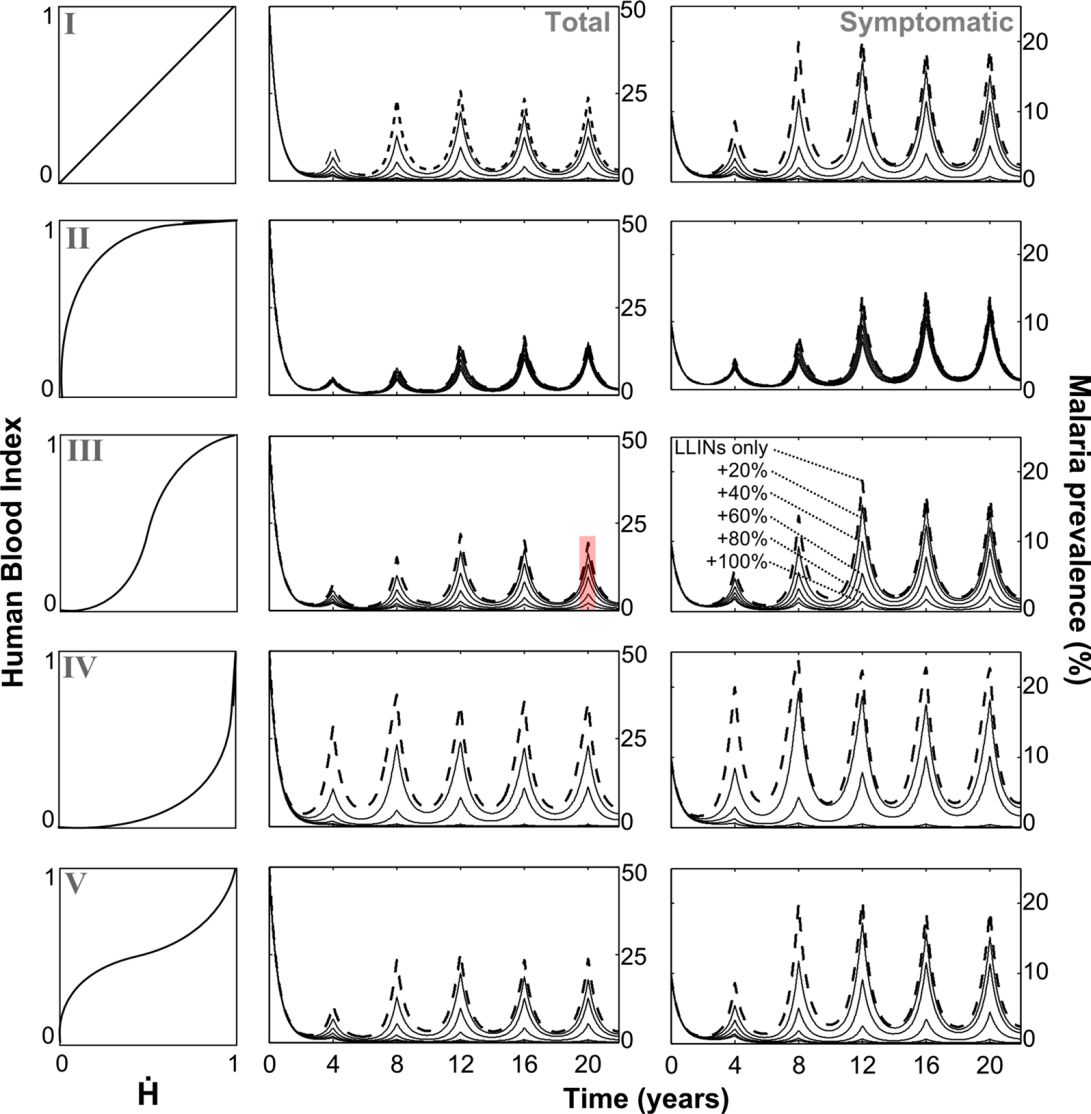
Vector ecology determines the impact of complementing LLINs with endectocide-treated cattle. *Left column* qualitatively distinct biting behaviours (Types I–V) arise from different functional relationships between the human blood index and the availability of humans relative to all potential blood hosts ($\dot{H}$). *Middle column* total malaria prevalence during the simulated control campaign. *Right column* symptomatic malaria prevalence during the campaign. *Dashed lines* LLINs as a stand-alone strategy; *solid lines* denote the additional control levels associated with complementing LLINs with 20, 40, 60, 80, and 100% coverage with endectocide-treated cattle. Oral treatment with 1.5 mg/kg fipronil is simulated to occur every 10 weeks (application frequency is fully explored in Fig. 3). Simulations were initiated at a steady state of (total) malaria prevalence of 50%. Parameter (*α*,*β*) values used to produce the different biting Types I–V, respectively, are: 1,1; 0.5,2; 2,2; 2,0.5 and 0.5,0.5. Results are shown above for $\dot{H}$ = 0.5. A sensitivity analysis of these parameters is shown in the Additional files. The *red stripe* shows how these results correspond to output in Fig. 3 in which it is shown how the peak infection level just prior to the final control distribution round is impacted by different endectocidal application frequency and coverage levels

Temporal malaria infection dynamics during a simulated transmission reduction campaign are shown in Fig. 2. Symptomatic and asymptomatic malaria prevalence during integrated vector management, combining LLINs with endectocide-treated cattle, is compared to LLINs alone for the different mosquito-biting ecologies. In all simulated LLIN-only control scenarios, malaria prevalence was significantly reduced. This reduction was most substantial for anthropophilic (Type II) vectors. The impact of LLINs was impeded (about half the effectiveness) when malaria was transmitted by zoophilic (Type IV) vectors. While the extent of this reduction attenuated over time between new LLIN applications, infection levels never fully returned to pre-control levels over the four-year time period of LLIN application frequency. Bed nets used as a standalone control tool were most effective at reducing malaria when mosquitoes were highly anthropophilic (displaying a Type II response). From an endemically stable 50% malaria prevalence (pre-control), new LLIN distribution drove infection cycles that peaked at 13% (just prior to new LLIN distribution) and troughed at 1% (just following new LLIN distribution). Conversely, this standalone strategy was least effective at controlling malaria when the local vectors were zoophilic and displaying a Type IV response (with prevalence cycling between 3 and 23%).

When specifically considering LLIN control of symptomatic infections (right-hand column of Fig. 2), rebounding prevalence exceeded pre-control levels after bed net efficacy was diminished. For some mosquito-biting ecologies, this rebound was substantial (up to a 2.5-fold increase relative to pre-control for a Type IV) as was the time period over which symptomatic prevalence exceeded pre-control levels (constituting 33% of a 4-year LLIN cycle for a Type IV mosquito).

For most mosquito-biting ecologies, even modest coverage levels of endectocide-treated cattle provided considerably improved malaria control efficacy. For an indiscriminate mosquito (with Type I response, i.e. the standard assumption of other malaria models that cater for non-human biting), the control combination including 80% coverage of endectocides maintained total malaria prevalence at below 0.2% during the 4-year cycle. Comparable gains in combined control were obtained for mosquitoes that displayed switched host preference as a function of relative host availability levels (prevalence was maintained below 1.7 and 0.2% for Type III and Type V, respectively). Intuitively, malaria transmitted by zoophilic mosquitoes (Type IV) experienced the greatest decline with endectocidal applications (maintained below 0.001%) whereas the least benefit to malaria control (almost negligible) was provided when mosquitoes were more anthropophilic (Type II). The qualitative nature of these results was not found to be sensitive to the parameters governing biting behaviour within the respective bounds of the biting Types (Additional file 1).

**Fig. 3:**
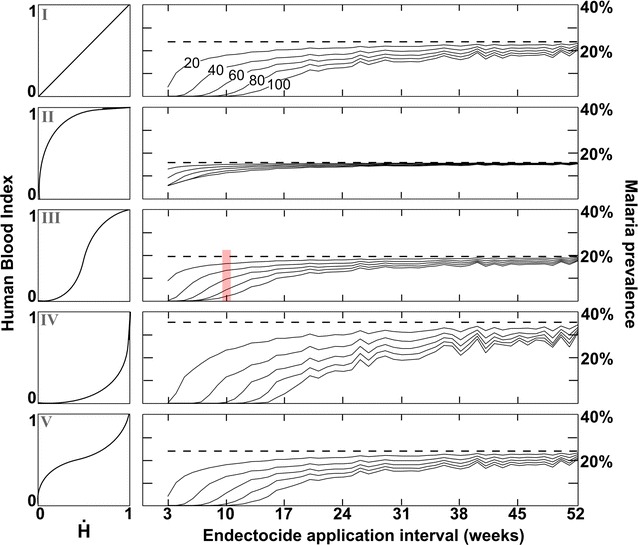
Malaria control efficacy as impacted by vector biting ecology and endectocide-treated cattle application frequency. Vector biting ecology is illustrated in the *left column*. Control levels are shown relative to LLINs alone (*dashed lines*) for increasing coverage levels of endectocide (*solid lines* denote 20, 40, 60, 80, and 100% coverage). Prevalence levels correspond with the final peak shown in the temporal dynamics of Fig. 1. Parameter (*α*,*β*) values used to produce the different biting Types I–V, respectively, are: 1,1; 0.5,2; 2,2; 2,0.5 and 0.5,0.5. Results are shown above for $\dot{H}$ = 0.5, and for different relative host availability in the Additional file 2. The *red stripe* shows how these results are related to output in Fig. 2, i.e. the stripe corresponds with the peak infection level just prior to the final control distribution round

A comprehensive analysis of endectocidal application frequency and coverage was conducted for the distinct vector-biting behaviours (Fig. 3). The peak malaria prevalence just prior to the sixth distribution round of LLINs is shown (i.e., corresponding with the final peak of Fig. 2) when using bed nets as a standalone strategy versus their integration with endectocide-treated cattle at varying coverage levels. The level of additional benefit achieved by integrating endectocides clearly diminishes with reduced coverage levels and reduced application frequency. Corresponding with the previous result, the greatest gains are achieved when controlling a zoophagic vector (Type IV) and only minor gains are achievable with an anthropophagic vector (Type II). Increasing coverage of endectocide-treated cattle has proportionately much greater impact when more frequent application is achievable. For most biting ecology Types, malaria prevalence can be decreased to such low levels as to represent complete control. For Type I this necessitates an endectocide application frequency of 11 weeks (100% coverage); a Type III mosquito needs a frequency of 7; 15 weeks for a Type IV and 10 weeks for a Type V.

## Discussion

In recent years, malaria control successes have brought to the fore plans for elimination, and rekindled hopes of eradication. Malaria control is largely dependent on mosquito management with LLINs or indoor residual spray (IRS). However, the spread of pyrethroid resistance challenges the sustainability of these current control mainstays. As well as new insecticides for IRS and LLINs, there is the need to reduce dependence on these two technologies and to find new ways to attack adult *Anopheles* malaria vectors. The current study constitutes the first theoretical examination of the combined use of endectocide-treated livestock with LLINs in order to assess the projected level of improvement in malaria control.

Targeting livestock-biting behaviour for controlling malaria mosquitoes is shown through simulation to have a potentially excellent synergy with LLINs for reducing malaria prevalence. Although it seems intuitive that adding more vector control tools should have the inevitable effect of improving upon efficacy, this is by no means a foregone conclusion. For example, previous theoretical studies have demonstrated that incorporating mosquito larvae breeding site management does not necessarily improve control projections when used as a complement to insecticidal nets unless breeding sites are already vanishingly rare and/or when nets have drastically compromised efficacy [38]. In terms of the most popular integrated vector management strategy in the world, LLINs and IRS, models have given conflicting results with some projections highlighting potential for interference between insecticidal modes of action actually reducing the benefit achieved with LLINs alone [39]. There is mounting evidence from the field to challenge the usefulness of this combination in some epidemiological/entomological settings [40–42], particularly when the additional control methods imply substantial additional costs.

In the current analysis, it was demonstrated that the epidemiological benefit of endectocide-treated cattle as an addition to LLINs is contingent on both the mosquito-biting ecology of local vectors and the intended purpose of the programme (being either morbidity reduction or elimination). Intuitively, endectocidal applications improve upon LLINs to the greatest degree when local vectors are more zoophilic (Type IV). Relatively modest frequencies of endectocidal application (in the order of three times per year) can substantially improve control efficacy when local vectors exhibit this behaviour. However, mosquitoes do not need to be particularly zoophagic for this control strategy to drastically improve projected control efforts—malaria spread by vectors that are indiscriminate in their host choice (Type I) is also controlled effectively with the integrated strategy. It should be emphasized that the current study is the first to explore the effects of combining these vector control methods, and as such, quite a simplistic model was used to present the results as transparently as possible. In order for the results described here to inform operational strategy, a more biologically detailed epidemiological model would be warranted, and this constitutes important future work. Further work could include adaptations of the mosquitoes to the reduced host availability resulting from LLINs. By reducing the proportional composition of humans, it may be posited that bed nets act to divert bites onto other host species—although, see Hii et al. [43] who do not show this effect. Importantly, however, this potential addition to future models would only have the effect of potentiating the control combination explored in the current analysis; it should be reiterated that the substantial benefits of this integrated strategy are likely only downplayed by the conservative estimates produced in this analysis.

To explore temporal dynamics of infection control, simple, exponential decay functions to describe the reduced efficacy of controls over time were included. Although more complicated functions have been discussed previously in the context of waning bed nets and IRS [39], exponential decay is the more popular method of incorporating this effect. Future work may be warranted to explore the impact of different decay functions for operational strategy, as more longitudinal efficacy data become available.

Waning malaria control inadvertently exacerbating symptomatic disease is the subject of discussion of several mathematical models. However, there is surprisingly little evidence of this phenomenon in practical reports from the field. In Africa, withdrawal of vector control has tended to lead to an eventual return to pre-control levels of infection prevalence [44], but changes in symptomatic cases are not reported. It is possible that this bounce-back is an artefact of models (including the one presented here). It is also possible that whatever causes the withdrawal or suspension of vector control may also weaken the surveillance needed to detect such a rebound, at least during the period immediately after withdrawal.

Regardless, if the objective of the programme is morbidity reduction, endectocide-treated cattle may additionally benefit a programme by reducing any temporary undesirable consequences of fluctuating levels of control on projected symptomatic infection rates. It can ameliorate disease when a control campaign is underway while also improving chances of achieving the goal of elimination. In other words, if one desired outcome of a given programme is morbidity reduction in the shorter term prior to elimination, there may be even greater justification for complementing LLINs with endectocide-treated livestock. With the current analysis, the first estimates of integrated vector management efficacy are provided for this novel pairing of interventions. Additionally, a framework is described for assessing the anticipated impact of these approaches on the control of a disease that is spread by vectors with increasingly recognized diverse feeding behaviours. Given the level of impact that this neglected behaviour is projected to have on disease intervention efficacy, generating empirical data to determine mosquito-biting behavioural Types constitutes an important future research goal.

## Additional files

**Additional file 1.** Sensitivity of temporal dynamics to parameters governing biting response Type. The sub-plots bounded by grey boxes are the parameter sets used in the main text figures to exemplify the five qualitatively different Types (labelled in top-left of these sub-plots). Temporal dynamics are shown for LLINs only (higher line in each sub-plot) as well as for LLINs + 100% coverage of endectocides (lower line in each sub-plot).

**Additional file 2.** Sensitivity of temporal dynamics to blood-host species composition. The column labels (1:3, 1:1 and 3:1) correspond with the ratio of humans: cattle. The row labels (on the right) correspond with the biting Type (I–V). Parameter (*α*,*β*) values used to produce the different biting Types I–V, respectively, are: 1,1; 0.5,2; 2,2; 2,0.5 and 0.5,0.5. As Fig. 2, the different lines within the sub-plot correspond with increasing levels (for lower lines) of endectocide used jointly with LLINs (bed nets as a standalone strategy are depicted in the top line of each sub-plot).
