# Supplementary figures and images for: Combining indoor and outdoor methods for controlling malaria vectors: an ecological model of endectocide-treated livestock and insecticidal bed nets
Source: Malar J. 2017 Mar 13;16:114. doi: 10.1186/s12936-017-1748-5 (PMC5347819; doi:10.1186/s12936-017-1748-5)

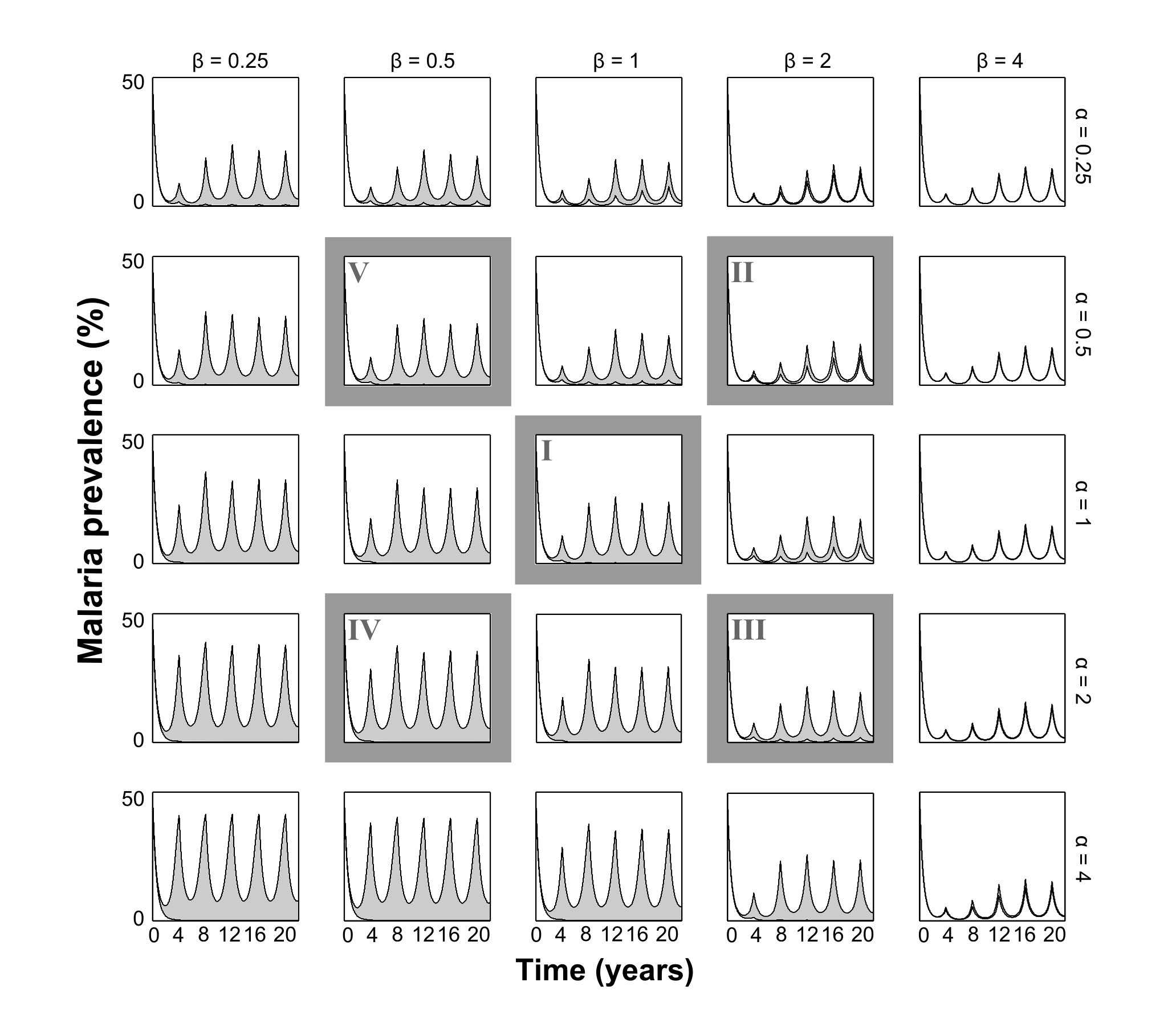

Supplement: Supplementary file 1 — Additional file 1. Sensitivity of temporal dynamics to parameters governing biting response Type. The sub-plots bounded by grey boxes are the parameter sets used in the main text figures to exemplify the five qualitatively different Types (labelled in top-left of these sub-plots). Temporal dynamics are shown for LLINs only (higher line in each sub-plot) as well as for LLINs + 100% coverage of endectocides (lower line in each sub-plot). [file 12936_2017_1748_MOESM1_ESM.png]

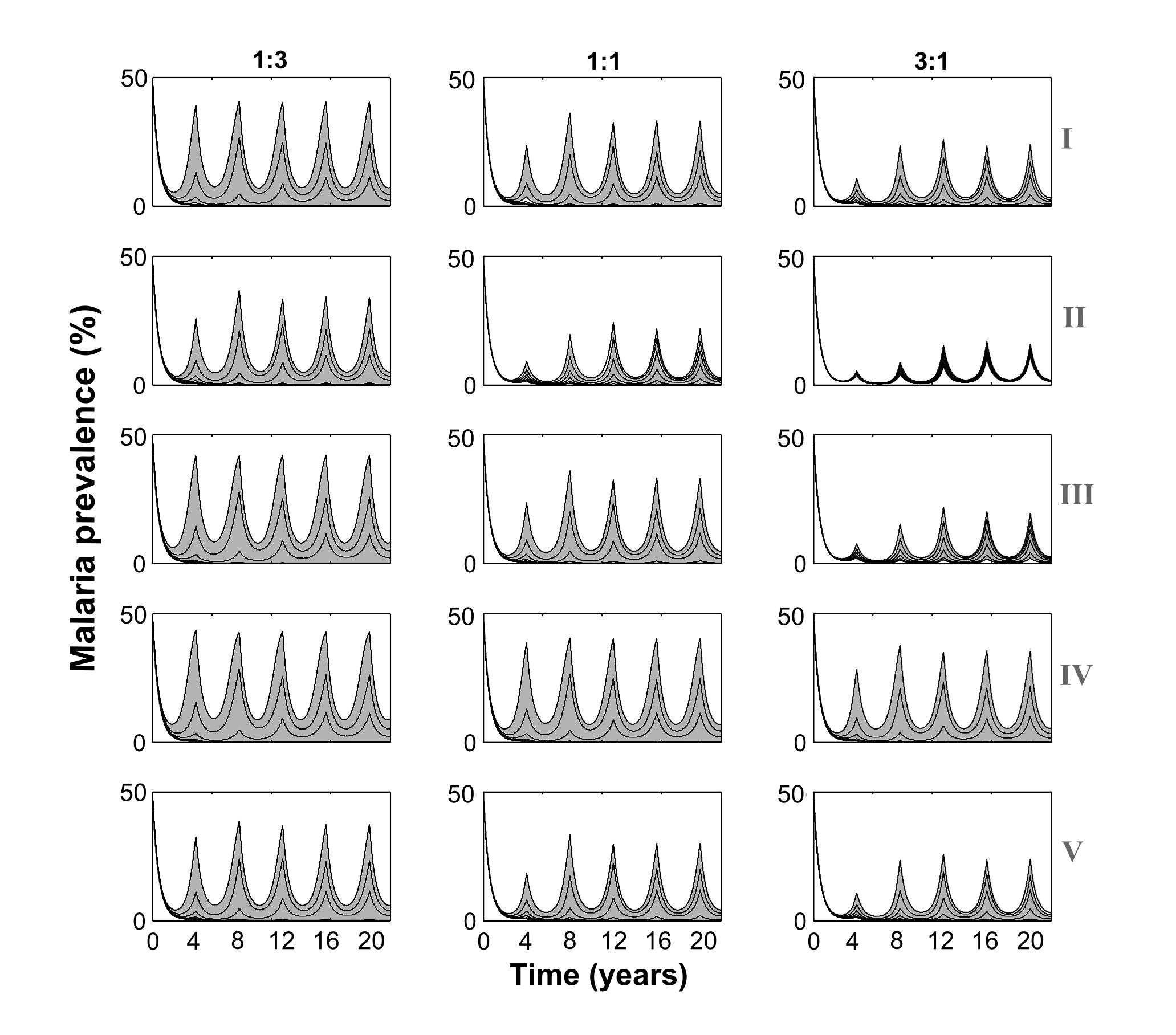

Supplement: Supplementary file 2 — Additional file 2. Sensitivity of temporal dynamics to blood-host species composition. The column labels (1:3, 1:1 and 3:1) correspond with the ratio of humans: cattle. The row labels (on the right) correspond with the biting Type (I–V). Parameter (α,β) values used to produce the different biting Types I–V, respectively, are: 1,1; 0.5,2; 2,2; 2,0.5 and 0.5,0.5. As Fig. 2, the different lines within the sub-plot correspond with increasing levels (for lower lines) of endectocide used jointly with LLINs (bed nets as a standalone strategy are depicted in the top line of each sub-plot). [file 12936_2017_1748_MOESM2_ESM.png]
